# Optimization of Extraction Process for Antidiabetic and Antioxidant Activities of Kursi Wufarikun Ziyabit Using Response Surface Methodology and Quantitative Analysis of Main Components

**DOI:** 10.1155/2017/6761719

**Published:** 2017-05-17

**Authors:** Salamet Edirs, Ablajan Turak, Sodik Numonov, Xuelei Xin, Haji Akber Aisa

**Affiliations:** ^1^The Key Laboratory of Plant Resources and Chemistry of Arid Zone, Xinjiang Technical Institute of Physics and Chemistry, Chinese Academy of Sciences, Urumqi 830011, China; ^2^State Key Laboratory Basis of Xinjiang Indigenous Medicinal Plants Resource Utilization, Xinjiang Technical Institute of Physics and Chemistry, Chinese Academy of Sciences, Urumqi 830011, China; ^3^University of the Chinese Academy of Sciences, Beijing 100039, China; ^4^State Scientifically-Experimental and Production Organization, Academy of Sciences of the Republic of Tajikistan, Dushanbe 734063, Tajikistan

## Abstract

By using extraction yield, total polyphenolic content, antidiabetic activities (PTP-1B and *α*-glycosidase), and antioxidant activity (ABTS and DPPH) as indicated markers, the extraction conditions of the prescription Kursi Wufarikun Ziyabit (KWZ) were optimized by response surface methodology (RSM). Independent variables were ethanol concentration, extraction temperature, solid-to-solvent ratio, and extraction time. The result of RSM analysis showed that the four variables investigated have a significant effect (*p* < 0.05) for *Y*_1_, *Y*_2_, *Y*_3_, *Y*_4_, and *Y*_5_ with *R*^2^ value of 0.9120, 0.9793, 0.9076, 0.9125, and 0.9709, respectively. Optimal conditions for the highest extraction yield of 39.28%, PTP-1B inhibition rate of 86.21%, *α*-glycosidase enzymes inhibition rate of 96.56%, and ABTS inhibition rate of 77.38% were derived at ethanol concentration 50.11%, extraction temperature 72.06°C, solid-to-solvent ratio 1 : 22.73 g/mL, and extraction time 2.93 h. On the basis of total polyphenol content of 48.44% in this optimal condition, the quantitative analysis of effective part of KWZ was characterized via UPLC method, 12 main components were identified by standard compounds, and all of them have shown good regression within the test ranges and the total content of them was 11.18%.

## 1. Introduction

With the rising morbidity of diabetes, the medicinal plants are widely used for the treatment and prevention of diabetes. The medicinal plants or natural products involve retarding the absorption of glucose by inhibiting the carbohydrate-hydrolyzing enzymes, such as *α*-glycosidase, and they are mostly safe and have good effect [1].* Geranium collinum* Steph. ex Willd. and* Hypericum Scabrum *Lnn. are distributed in central Asia and they are recorded in the “Chinese pharmacopoeia” [2] and “kazakh medicine” [3]. Different types of* Geranium* are widely used for treatment of diabetes in Tajik, Chinese, and Mongolian traditional medicine [4].* G. collinum* has been used for the treatment of rheumatism, gout, dysentery, and external and internal bleeding, as well as in the treatment of skin wounds, eczema, scabies, tenosynovitis, and pruritus [5]. Aerial part of* H. scabrum* has shown significant antidiabetic and antioxidant activities [6]. Under optimization of the process of extraction and evaluation of antidiabetic potent activity (Supplementary Material, Table S1, in Supplementary Material available online at https://doi.org/10.1155/2017/6761719), the best proportion was found to be 7 to 3 and we named the prescription Kursi Wufarikun Ziyabit (KWZ). But the better extraction method for obtaining high yield, polyphenol content, and active parts was still unknown and the chemical constituents of the active part were still confused.

*G. collinum* is the Geraniaceae of the genus* Geranium* and distributed in the east, west Asia, central Asia, central Europe, and Xinjiang of China [7]. Species of* Geranium* was the important herbal medicine in folk and modern society. Recently researches show that this kind of plant has a variety of biomedical activities such as antioxidant activity, anti-inflammatory activity, use against diarrhea, ulcer healing, reducing blood sugar, and prevention and treatment of diabetes complications [8–15]. The main components of the species include polyphenols, flavonoids, organic acid, and terpenoids [16].

*H. scabrum* is one of the species of genus* Hypericum*;* Hypericum* species also have many biological activities such as antiviral, wound healing, antioxidant, antimicrobial, antifungal, anxiolytic, and anticonvulsant activities [6, 17]. Beside this, some species of* Hypericum* also show effect in the treatment of burns and gastrointestinal diseases and as antidepressant. The species contain a variety of compounds such as tannins, flavonoids, hyperforin derivates, and essential oil [18]. Recently researches show these type of medical plants have been a rich source of hypoglycemic components [19].

With the development of people's living standard and aging of population, the incidence of diabetes has risen sharply. According to the World Health Organization (WHO), approximately 350 million individuals suffer from diabetes mellitus (DM) and this may double by 2030 [20]. Therefore, the prevention and treatment of diabetes are becoming more and more important in the field of clinical research. At present, the world has developed more than 100 kinds of drugs for resistance to high blood sugar and to reduce fasting and postprandial blood glucose concentrations to normal, healthy levels without hypoglycaemia are one of the main goals of drug development for diabetes [21]. Protein tyrosine phosphatase-1B (PTP-1B) and *α*-glucosidase are the most efficient inhibitors of carbohydrate-hydrolyzing enzymes, serving as a most common therapeutic target for type 2 diabetes mellitus (T2DM), as well as being beneficial in the management of blood glucose in patients with T2DM, insulin resistance, and obesity [22, 23]. At present, PTP-1B and *α*-glucosidase have received significant attention as the important drug target for T2DM [24].

Response Surface Methodology (RSM) is the mathematical and statistical analysis method for optimizing extraction processes in order to obtain the desirable responses, and the Box-Behnken has become the most popular design tools for estimates of the effects of individual variables [25]. For this response, the key variables and experimental design can be found out before applying the RSM. The objective of this study was to determine the optimal extraction process of bioactive components, to identify the main compounds, and to quantitative analyze the major compounds in the effective parts for KWZ.

Over the past years, the qualitative and quantitative analysis of major components in medicinal plants were widely used by high performance liquid chromatography (HPLC) method because of its convenience and efficiency [26–28]. However, the quantitative analysis of plant extracts by this method need longer operation; meanwhile it requires about one or more hours for a single run. In recent years, ultraperformance liquid chromatography (UPLC) has emerged as a viable technique for qualitative and quantitative analysis of natural products [29].

At present, this prescription is in private consumption for diabetic mellitus. However, there is no comprehensive guideline on the medical application, qualitative and quantitative analysis of active ingredients, and the best way to get active parts. Therefore, according to the source of folk prescription and hypoglycemic activity, the best ratio of 7 : 3 was determined. Then, the experiment was designed to find the best extraction method using response surface methodology and quantitative analysis of active parts with the lowest cost, high yield, and strong hypoglycemic activity.

In this paper, the optimal extract conditions for prescription materials were found. The influence of the extraction conditions including ethanol concentration, temperature, sample-to-solvent ratio and extraction time, and responses were observed including extraction yield, polyphenol content, antidiabetic activity (PTP-1B and *α*-glucosidase activity), and antioxidant activity. 12 kinds of main components in effective parts for KWZ were identified and quantitative analyzed by UPLC.

## 2. Materials and Methods

### 2.1. Plant Materials

Root of* G. collinum *and aerial parts of* H. scabrum* were collected from Takob village of the Republic of Tajikistan (38.5357500 N, 68.7790500 E, and 2000 m above sea level, Tajikistan). The plants were identified by Professor Yusuf Nuraliev from Avicena's Institute of Medicine and Pharmacology of the Republic of Tajikistan. Voucher specimens (Barcode:* G. collinum *WY01053,* H. Scabrum *WY01054) were deposited at the Herbarium of the Key Laboratory of Plant Resources and Chemistry of Arid Zone, Xinjiang Technical Institute of Physics and Chemistry, Chinese Academy of Sciences.

### 2.2. Chemicals and Reagents

All of the solvents used for chromatographic analysis were of HPLC grade (Merck, Germany), solvents used for extract were of analytical grade (Baishi Chemical Co. Ltd., Tianjin, P. R. China), and water was double distilled. PTP-1B (human, recombinant) was expressed and purified in the Key Laboratory of Plant Resources and Chemistry of Arid Zone, Uygur drug activity screening room, Xinjiang Technical Institute of Physics and Chemistry, Chinese Academy of Sciences, and stored in a −80°C.* P*-nitrophenyl phosphate (*p*NPP), *α*-glucosidase, 4-N-trophenyl-a-D-glucopyranoside (*p*NPG), ABTS, and DPPH were purchased from Sigma Aldrich Co., LLC (St. Louis, Missouri, USA). The standard compounds for content determination were purchased from Beijing Century Aoke Biology Research Co., Ltd. (Beijing, China) or Shanghai PureOne Bio Tech Co., Ltd. (Shanghai, China) and their purity were higher than 98%.

### 2.3. Extraction Process

In this study, powdered part of prescription medicine materials was accurately weighed in accordance with the ratio (root of* G. collinum*: aerial parts of* H. scabrum* = 7 : 3) 5 g and placed in round-bottom flask and then was extracted (reflux extraction) using ethanol/water as solvent in different concentration (30, 50 and 70%) (Supplementary Material, Table S2), different temperature (60, 70 and 80°C) (Supplementary Material, Table S3), different sample-to-solvent ratio (1 : 10, 1 : 20 and 1 : 30* w/v*) (Supplementary Material, Table S4), and different extraction time (2, 3 and 4 h) (Supplementary Material, Table S5). All experiment materials were extracted 3 times (Supplementary Material, Table S6) for all experiments. Filtered extracts were evaporated in a rotary vacuum evaporator and then dried at 45°C for 24 h. Then the evaporated extracts were further dried using freeze-drying (FDU-2100; Eyela, Tokyo, Japan) at −80°C for 36 h. Then the dried matter was powdered, weighed, and packed in zip pack bags, stored at 4°C for further analysis.

### 2.4. Determinations

#### 2.4.1. Extraction Yield (EY)

The extraction yield was calculated as the weight (g) of KWZ in the extract compared to that in the dried raw material, expressed as a percentage, as shown in

(1) $$\begin{array}{c} EY\left(\;\%\;\right)=\frac{W_{1}}{W_{2}}\times 100, \end{array}$$

where *W*_1_ is the weight of KWZ after the extraction (g) and *W*_2_ is the weight of KWZ before extraction (g).

#### 2.4.2. Total Polyphenol Content (TPC)

Total polyphenols in the extracts were estimated according to the determination of tannin content method [3]. The extracts were diluted to the concentration of 10 *μ*g/mL and aliquots of 0.5 mL placed in 25 mL brown volumetric flask, and add 1 mL phosphomolybdic acid test solution and 11.5 mL water and 15% NaHCO_3_ volume to the scale. After 30 min incubation at room temperature, the absorbance of the mixtures was measured at 760 nm by using UV/Vis spectrophotometer (Shimadzu, Japan) against a blank sample. Gallic acid (GA) was used as the standard (*R*^2^ = 0.9995). The results were calculated as the microgram of gallic acid equivalents per milliliter of liquid extracts compared to the total sample concentration, expressed as a percentage, as shown in

(2) $$\begin{array}{c} TPC\left(\;\%\;\right)=\frac{C_{1}}{C_{2}}\times 100, \end{array}$$

where *C*_1_ is the microgram of gallic acid equivalents per milliliter of liquid extracts (*μ*g/mL) and *C*_2_ is the total sample concentration (*μ*g/mL).

#### 2.4.3. PTP-1B Assay

In this study, the 29 extracts were tested to determine the activity of PTP-1B using* p*NPP (p-nitrophenyl phosphate disodium salt) as a substrate. In each well in the 96-well microtiter plate (final volume: 200 *μ*L) we added 178 *μ*L PBS buffer (20 mM HEPES, 150 mM NaCl, 1 mM EDTA, pH 7.4), 1 *μ*L PTP-1B (0.115 mg/mL), and 1 *μ*L test sample (or Dimethyl sulfoxide as a blank), then mixed well for 10 min, and added 20 *μ*L 35 mM substrate* p*NPP. Thereafter, the plate was incubated without light at 25°C for 20 min, and the reaction terminated with 10 *μ*L of 3 M sodium hydroxide. The absorbance values were measured by SpectraMax MD5 Microplate Reader (Molecular Devices, USA) at 405 nm, with the system without enzyme solution in a blank. The inhibition rate (IR) was calculated using the following equation:

(3) $$\begin{array}{c} IR\left(\;\%\;\right)=\frac{P_{C}-P_{S}}{P_{C}}\times 100, \end{array}$$

where *P*_*C*_ is the absorbance of the control and *P*_*S*_ is the absorbance of the sample.

#### 2.4.4. *α*-Glucosidase (*α*-Glu) Assay

*α*-Glu inhibitory activity was determined in a 96-well plate using* p*NPG as a substrate. In each of the 96 wells in a microtiter plate (final volume: 100 *μ*L) we added 68.5 *μ*L 0.1 mM phosphate buffer (pH 6.8), 1.5 *μ*L enzyme solution (0.2 U/mL *α*-glucosidase in a phosphate buffer), and 5 *μ*L test sample (or Dimethyl sulfoxide as a blank) mixed and incubated at room temperature for 10 min, and then 25 *μ*L 20 mM* p*NPG was added. Thereafter, the plate was incubated at 37°C for 20 min. The absorbance values were measured by SpectraMax MD5 Microplate Reader at 405 nm, with the system without enzyme solution in a blank. The inhibition rate (IR) was calculated using the following equation:

(4) $$\begin{array}{c} IR\left(\;\%\;\right)=\frac{G_{O}-G_{S}}{G_{O}}\times 100, \end{array}$$

where *G*_*O*_ is the absorbance of the blank and *G*_*S*_ is the absorbance of the sample.

#### 2.4.5. Antioxidant Activity

The antioxidant ability of extracts was determined by ABTS and DPPH. The ABTS scavenging assay was performed according to Hui's method [30] with a slight modification. 7 mM ABTS solution that dissolved in 20 mM sodium acetate (PH 4.5) was reacted with 2.45 mM potassium persulphate to generate ABTS^+^ radical cation, and the mixture was kept in dark room temperature for 12–16 h before use. After that, the ABTS^+^ solution was diluted with ethanol to an absorbance of 0.70 ± 0.2 at 734 nm. 16 *μ*L of test sample was mixed with 184 *μ*L of ABTS solution in 96 wells' plate, and absorbance values were measured by SpectraMax MD5 Microplate Reader after 5 min, and the system without ABTS solution was used as the blank.

DPPH test was performed according to Zhang's method [31] with a slight modification. The sample (100 *μ*L) was mixed with 0.2 mM DPPH solution in 100 *μ*L. The reaction was incubated in the dark for 30 min at the room temperature. The absorbance was measured at the wavelength of 515 nm, and the inhibition rate was calculated. The positive controls in the ABTS and DPPH tests were vitamin C. The inhibition rate (IR) was calculated using the following equation:

(5) $$\begin{array}{c} IR\left(\;\%\;\right)=1-\frac{A_{O}-A_{1}}{A_{O}}\times 100, \end{array}$$

where *A*_*O*_ is the absorbance of the blank and *A*_1_ is the absorbance of the sample.

### 2.5. Experimental Design

In order to determine the effects of extraction parameters and optimize conditions for various responses RSM optimization method was applied. Box-Behnken design (BBD) consisted of 29 randomized runs with 5 replicates at the central point. The effects of extraction independent parameters (ethanol concentration (*X*_1_, %), temperature (*X*_2_, °C), sample-to-solvent ratio (*X*_3_,* w/v*), and extraction time (*X*_4_, h)) were encoded as −1, 0, and +1. The coded and uncoded variables are listed in Table 1. The levels of these responses (extraction of yield (*Y*_1_), TPC (*Y*_2_), PTP-1B inhibition rate (*Y*_3_), *α*-glucosidase inhibition rate (*Y*_4_), and ABTS inhibition rate (*Y*_5_)) were selected based on our preliminary study. The model equation for the response (*Y*) to the four independent variables (*X*_1_, *X*_2_, *X*_3_, and *X*_4_) is given in the following equation:

(6) $$\begin{array}{c} Y=b_{o}+\sum b_{i}X_{i}+\sum b_{ii}X_{ii}^{2}+\sum b_{ij}X_{i}X_{j}. \end{array}$$

### 2.6. Extraction and Purification of KWZ

The air-dried and powdered aerial part of prescription materials (500 g) was extracted with 50% ethanol (1 : 20 w/v) three times for 3 h at 70°C. The extract was concentrated under vacuum to 1.02 g/mL. Concentrated extractive (250 mL) was purified with a column of HPD300 macroreticular resin (50 mL) and after that washed with 150 mL of distilled water, then first eluted with 100 mL of 30% ethanol, and after that eluted with 150 mL of 70% ethanol. Thereafter, the eluted parts of 30% ethanol and 70% ethanol were combined, and then they were concentrated and dried using freeze-drying (FDU-2100; Eyela, Tokyo, Japan) at −80°C for 36 h. The dried matter was powdered, weighed, and packed in zip pack bags, stored at 4°C for further analysis.

### 2.7. Sample Preparation and Optimization of UPLC Chromatographic Condition

The purified prescription was dissolved in 50% methanol/H_2_O and filtered through 0.22 *μ*m nylon membrane microfilters (Shimadzu-GL, Japan). The chromatographic analysis was achieved using a Waters ACQUITY Ultraperformance Liquid chromatography (UPLC) with a photodiode detector (PDA) (Waters, Milford, MA, USA). Reversed-phase separation was performed on an ACQUITY UPLC BEH Shield RP18 (2.1 × 100 mm, 1.7 *μ*m, Waters, Milford, MA, USA) column at 35°C. Mobile phases comprised (A) 0.2% formic acid in water and (B) acetonitrile. The sample was injected (2 *μ*L injection volume) onto the column and eluted at a flow rate of 0.25 mL/min according to the following gradients: initial 5.0% B; 0.0–3.0 min/5.0–6.0% B; 3.0–14.0 min/6.0–7.0% B; 14.0–15.0 min/7.0–9.5% B; 15.0–15.5 min/9.5–10.0% B; 15.5–20.0 min/10.0% B; 20.0–20.5 min/10.0–11.0% B; 20.5–35.0 min/11.0% B; 35.0–36.0 min/11.0–11.5% B; 36.0–43.0 min/11.5% B; 43.0–57.0 min/11.5–16.0% B; 57.0–72.0 min/16.0–21.0% B; 72.0–78.0 min/21.0–24.0% B; 78.0–84.0 min/24.0–30% B; 84.0–90.0 min/30.0–38.0% B; 90.0–93.0 min/38.0–60.0% B; 93.0–94.0 min/60.0–100.0% B. Ultraviolet detection was set to 254 nm.

### 2.8. Data Analysis

In this study, 29 experiments that were planned with the BB design were carried out for building quadratic models, with 5 replications of the center points to estimate the experimental errors. The weighed extracts were calculated with extraction yield using Microsoft Excel™ 2016 (Microsoft, USA) and used for antidiabetic activities and antioxidant activities analysis. The data were calculated with inhibition rate and half-inhibition concentration using SPSS 19.0 (SPSS Inc., Chicago, IL, USA). The Design Expert v.8 trial (Stat-Ease, Minneapolis, Minnesota, USA) was used for data analysis, regression model building, and experimental design and to predict the optimal processing parameters.

## 3. Results and Discussion

### 3.1. Effects of Extraction Parameters on Extraction Yield (EY)

**Figure 1:**
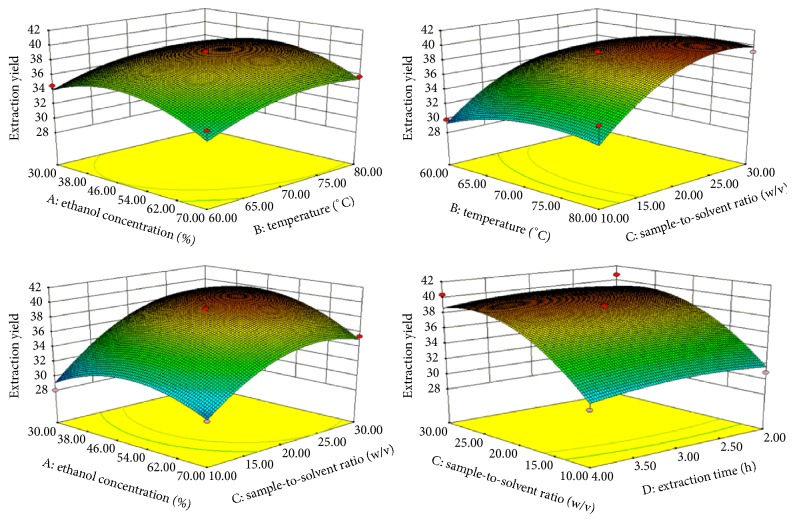
Response surface plot for the effects of investigated parameters on the EY.

It is well known that solvent concentration, temperature, sample-to-solvent ratio, and extraction time were factors that most influence the yield of extractions in plant extracts. In this study, the EY of 29 designed experiments in the current BBD are shown in Table 2. The regression equation was shown in Table 3 with *R*^2^ = 0.9120. In a general way, high *F* values with low *P* values lead to more significant correspondence amongst independent variables. *X*_2_, *X*_3_, *X*_1_^2^, *X*_2_^2^, and *X*_3_^2^ were significant (*p* < 0.05), whereas *X*_1_, *X*_4_, *X*_4_^2^, *X*_1_*X*_2_, *X*_2_*X*_3_, *X*_1_*X*_4_, *X*_2_*X*_3_, *X*_2_*X*_3_, *X*_2_*X*_3_, *X*_2_*X*_4_, *X*_2_*X*_4_, and *X*_3_*X*_4_ were not significant (*p* > 0.05).  Figure 1 shows the 3D surface plots of the yields as influenced by each extraction condition. The most important factor influencing the EY is *X*_3_. EY in examined samples decreased with a low *X*_3_, increased with more *X*_3_. This is probably because the less solvent is leading to incomplete extraction, whereas too much solvent can result in a high experimental cost [32].

In this part, high *X*_3_ would improve the EY, as well as the high polyphenol content, and good results of antidiabetic activities and wally antioxidant activity would be got about 1 : 20 g/mL *X*_3_. Hwang et al. reported that high extraction yield and high polyphenol content should be obtained by longer extraction time and higher solvent volume [33]. In addition, some extraction yields would be increased by high *X*_2_, but with high value of *X*_2_ causing loss of polyphenol content and decrease of antidiabetic activities and antioxidant activity. Moreover, experimental results of response variables were similarly influenced by *X*_4_ as the change of time in the medium. Jeong et al. [32] and Zheng et al. [34] testified that factors with the most influence for high extraction yield and high polyphenol content are longer extraction time and high solvent volume. According to the experimental results, the EY of KWZ continued to increase as *X*_3_, perhaps because the solvent volume increased diffusion and enhanced desorption of active part from prescription. Optimal conditions for the highest EY of 40.84% were derived at *X*_1_ = 48.80%, *X*_2_ = 74.29°C, *X*_3_ = 1 : 25.09 g/mL, and *X*_4_ = 3.04 h (Table 4).

### 3.2. Effects of Extraction Parameters on Total Polyphenol Content (TPC)

**Figure 2:**
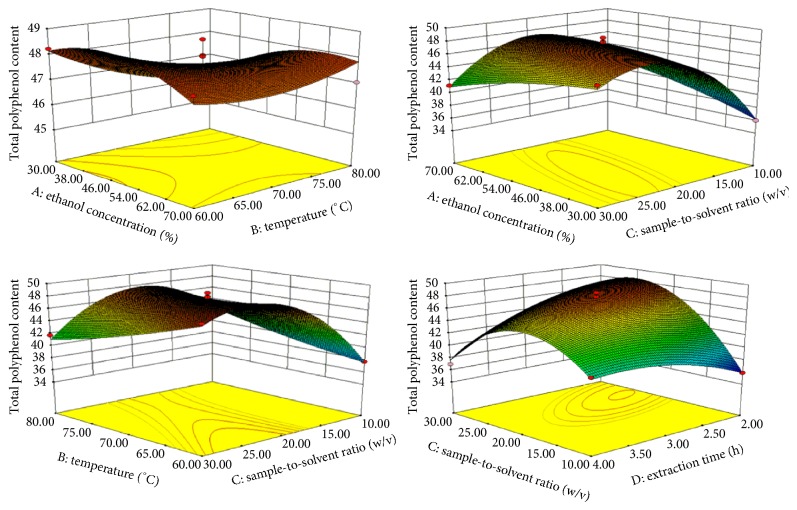
Response surface plot for the effects of investigated parameters on the TPC.

The effects of different extraction parameters on the TPC are shown in Table 2. The analysis of variance (ANOVA) on the experimental data with *R*^2^ = 0.9793 and the polynomial equation are shown in Table 3. Influencing factors on TPC are *X*_3_ > *X*_4_ > *X*_2_ > *X*_1_, respectively. It can be seen that *X*_1_ and *X*_3_ were the most significant factors for TPC (*p* < 0.05), the increase of *X*_1_ and *X*_3_ caused an increase of TPC, and they decrease with an increase of *X*_2_ and *X*_4_. The relationship between TPC and both factors is shown in Figure 2, and when *X*_1_ increased from 38% to 54% and *X*_3_ increased from 1 : 15 g/mL to 1 : 25 g/mL, TPC value also increased about 48%. Table 4 shows the predicted values of the maximum TPC extraction that 48.44%, when *X*_1_, *X*_2_, *X*_3_, and *X*_4_ volume were 50.11%, 72.06°C, 1 : 22.73 g/mL, and 2.93 h, respectively.

Although *X*_1_ and *X*_3_ directly affect the TPC, *X*_2_ is also one of the main factors that influence the polyphenol content. Low *X*_2_ was ineffective in the extraction process to release bioactive substance from KWZ, causing destruction of the activity by high *X*_2_. Mašković et al. [35] reported that the antioxidant activity and the content of polyphenols of orange fruit can be improved by using 40–50% ethanol concentration, 50–60°C temperature, and 120–130 min extraction time. Results showed that higher TPC (>45%) could be obtained with the higher *X*_3_ (1 : 15–1 : 25 g/mL) and *X*_4_ (>2.5 h) and lower *X*_1_ (45–55%), in comparison with higher temperature and higher sample-to-solvent ratio.

### 3.3. Antidiabetic Activities of Extracts

In this study we have determined two responses (PTP-1B and *α*-glucosidase) for antidiabetic activities of 29 designed experiments. Protein tyrosine phosphatase-1B (PTP-1B) and *α*-glucosidase (*α*-Glu) are two of the most commonly insulin resistance states for T2DM and received the significant attention during the recent years [36]. PTP-1B is the main negative regulator of insulin signaling pathway, and *α*-glucosidase inhibitor is the preferred drug for the treatment of T2DM [37, 38].

**Figure 3:**
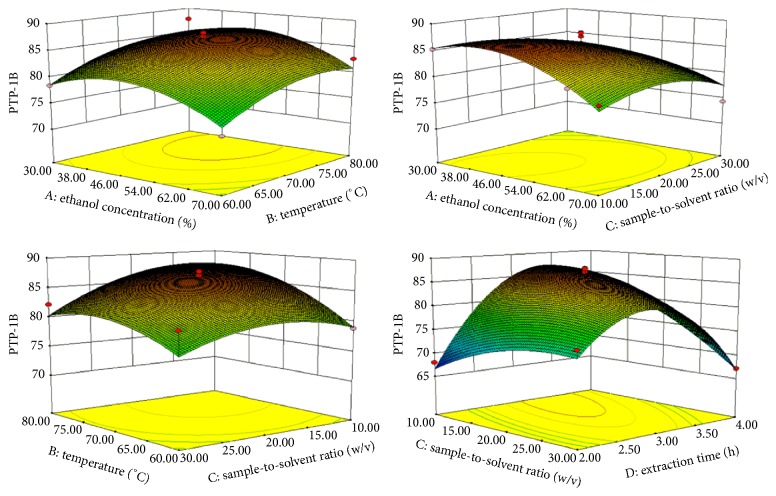
Response surface plot for the effects of investigated parameters on the PTP-1B inhibition rate.

The quadratic relationship between the PTP-1B and extraction variables had a good regression coefficient of *R*^2^ = 0.9076. Then PTP-1B inhibition rates of 29 design experiments are shown in Table 2, ANOVA on the experimental data shown in Table 3. Influencing factors on PTP-1B are *X*_2_ > *X*_4_ > *X*_3_ > *X*_1_, respectively. The PTP-1B inhibition rate increased as *X*_2_ increased from 70°C to 75°C and *X*_4_ increased about 3 h (Figure 3). Optimal conditions for a maximum PTP-1B inhibition rate of 86.21% were derived at *X*_1_ = 52.10%, *X*_2_ = 73.80°C, *X*_3_ = 1 : 21.84 g/mL, and *X*_4_ = 2.95 h (Table 4).

**Figure 4:**
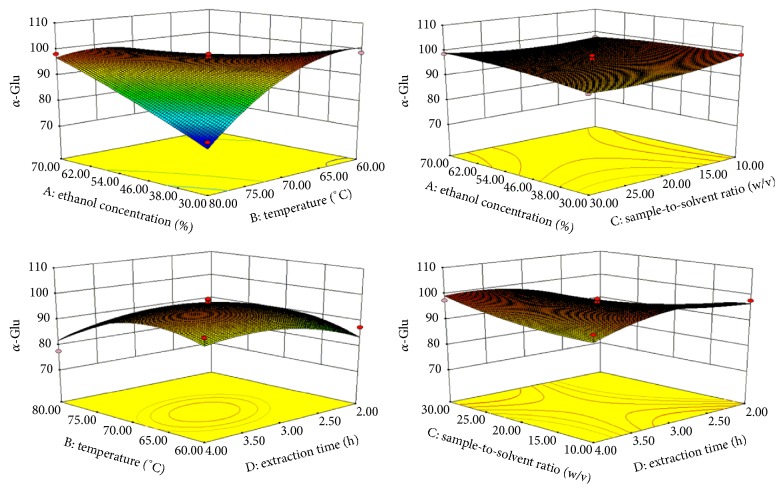
Response surface plot for the effects of investigated parameters on the *α*-glucosidase inhibition rate.

*R* ^2^ for the regression coefficient of *α*-Glu was 0.9125 (Table 3). The predicted optimum condition for *α*-Glu was 96.56% when *X*_1_, *X*_2_, *X*_3_, and *X*_4_ were 47.57%, 69.74°C, 1 : 22.22 g/mL, and 3.03 h, respectively (Table 4). It can be seen from the quadratic equation that *X*_2_ and *X*_4_ were the most significant factors for *α*-Glu inhibition rate (*p* < 0.05) (Table 3). The *α*-Glu inhibition rate observed with increasing of *X*_2_ and *X*_4_ was about 65–70°C and 2.30–3.30 h, respectively (Figure 4).

As a whole, the most influencing extraction factors for antidiabetic activities are *X*_2_ and *X*_4_. As can be seen in Figures 3 and 4, the up and down of *X*_2_ and *X*_4_ will also affect the activities of antidiabetics. The low *X*_2_ and short *X*_4_ may have effect on the extraction of active substances completely, and the high *X*_2_ and *X*_4_ maybe cause the loss of active substances. Mohamed et al. reported that they found the most active inhibition of *α*-glucosidase and alpha-amylase with 50% ethanol concentration [39]. The highest inhibition rates of KWZ were observed at higher *X*_2_ and *X*_3_. *X*_2_, *X*_3_, and *X*_4_ have shown effects on inhibition rate by their interaction. Then, the present extraction studies demonstrated that appreciable antidiabetic activities would be achieved in about 45–55%  *X*_1_ and about 65–75°C *X*_2_ and 2.30 −3.30 h *X*_4_, respectively.

### 3.4. Antioxidant Activities of Extracts

**Figure 5:**
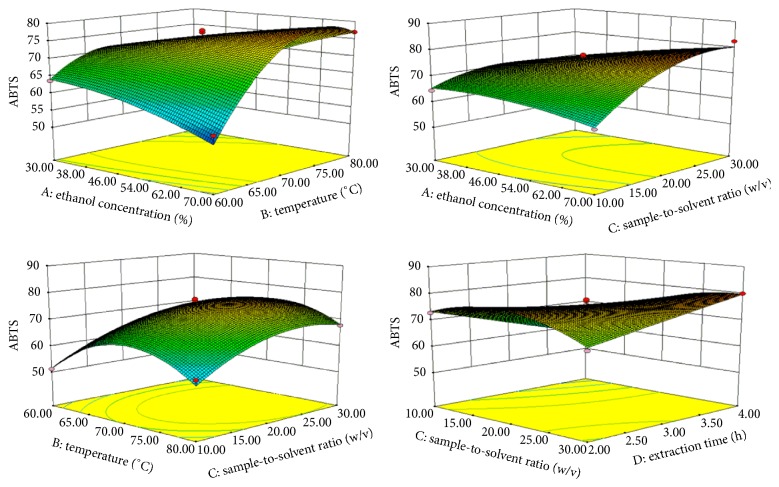
Response surface plot for the effects of investigated parameters on the ABTS inhibition rate.

KWZ not only has hypoglycemic activity but also has a higher antioxidant activity (Tables 2 and 5). The antioxidant activity of KWZ was determined with ABTS and DPPH assays. Influencing factors on ABTS are *X*_3_ > *X*_2_ > *X*_1_ > X_4_, respectively. *R*^2^ = 0.9709 and quadratic equation for the regression coefficient of ABTS are shown in Table 3. The optimum predicted point of ABTS inhibition rate was 77.68% as independent variables being *X*_1_ = 52.70%, *X*_2_ = 71.50°C, *X*_3_ = 1 : 22.38 g/mL, and *X*_4_ = 3.01 h (Table 4). 3D surface plots of ABTS show that the most influencing extraction factors on antioxidant activity are *X*_2_ and *X*_3_ (Figure 5). Also, the ABTS inhibition rate increased at *X*_2_ and *X*_3_ around 70–75°C and 1 : 20–1 : 25 g/mL, respectively. On the other hand, ABTS inhibition rate decreased at *X*_2_ above 75°C and *X*_3_ above 1 : 25 g/mL.

In this work, KWZ is enriched in polyphenols and exhibited an excellent antioxidant activity with ABTS and DPPH. The ABTS and DPPH test show the results (Tables 2 and 5). Since the test of ABTS and DPPH was all attributed in free radical scavenging activity and the trend of their results was very similar, in order to avoid repeated analysis with the result of the experiments, no ANOVA was carried out on the DPPH.

On the whole, the two parameters were both inhibited by KWZ. Then, Table 5 showed that the IR values of DPPH ranged from 78.71 to 97.65% with the different extraction samples, comparable to 88.98 ± 0.42% of *V*_*c*_. These results indicated that KWZ have a good free radical scavenging capacity, and the antioxidant capacities of the 29 designed experiments were significantly different. Observing ABTS and DPPH test results of 29 designed experiments, we found the antioxidant activities of the extracts are susceptible to temperature change, as well as the antidiabetic activity. In the case of high *X*_2_, some polyphenols would be degraded and their yields would be reduced, then causing a decrease in the antioxidant activity. Low *X*_2_ affects the release of polyphenols, which affects the decrease of antioxidant activity. So, polyphenol content and its antioxidant activity have been improved by using a 50%  *X*_1_ and 60°C *X*_2_ and above 60%  *X*_1_ would decrease the antioxidant activity [30]. In our work, the good antioxidant ability of ethanol extracts of KWZ determined by ABTS and DPPH and the obvious effects of temperature and sample-to-solvent ratio on response change were observed. This information was used to test the accuracy of the model's prediction of optimum response values by comparing it with the optimum levels obtained by the RSM optimization. The optimum predicted point of ABTS inhibition rate was 77.68%.

### 3.5. The Quantitative Analysis on Effective Part of KWZ

#### 3.5.1. Method Validation of UPLC Analysis

**Figure 6:**
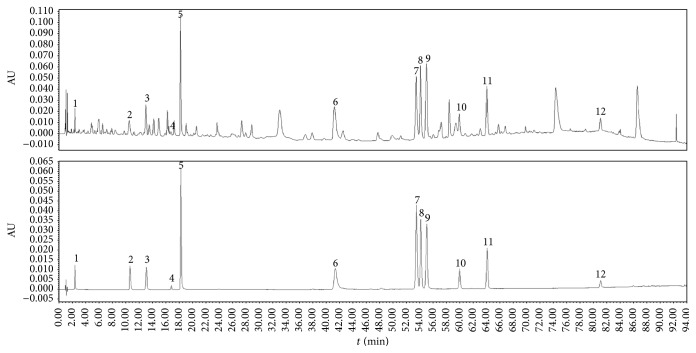
UPLC chromatograms of the KWZ and mixed standard.

According to the early studies on the chemical constituents of the two medicinal plants, the standard compounds were purchased and used for quantitative analysis. UPLC chromatogram of purified prescription and 12 compounds of mixed standard is shown in Figure 6. First, the method of UPLC analysis on purified prescription was validated with precision, repeatability, and stability tests. Intraday precision and repeatability as well as interday stability of the UPLC method were determined and expressed by the relative standard deviations (RSD) value of the average relative retention times (RRT) and relative peak areas (RPA) of the 12 peaks, with the peak that had a high content, a stable peak area, and a good shape at retention time (*t*_*R*_) of 18.2 min in the chromatogram as reference peak (peak 5). The intraday precision variation of the RRT and RPA of the 12 peaks was less than 0.20% and 2.00%, respectively. The stability test was evaluated by analysis of the same sample solution on two consecutive days at different time intervals (0, 3, 6, 12, 18, 24, 36, and 48 h), and the RSD values of RRT and RPA of the 12 peaks were below 0.30% and 2.00%, respectively. It means the sample solution was stable within 48 h. The repeatability test was calculated by analysis of six independently prepared solutions of the same sample. The RSD values of RRT and RPA did not exceed 0.30% and 2.00%, respectively. The results of the precision, stability, and repeatability tests are shown in Table 6.

#### 3.5.2. Quantitative Determination of Twelve Components on Effective Part of KWZ

Twelve components were identified in the effective part of KWZ (Figure 6). To determine the content of the components, firstly, the linearity of this method was evaluated. Standard solutions were prepared by diluting specific volume of standard to get several concentrations. The regression equations of the 12 components were calculated in the form of* y = ax + b*, where* y* and* x* were peak area and concentration, respectively (Supplementary Material, Fig. S1–S12). The contents of the 12 components were calculated by one point external standard method. Retention times, component names, regression equations, contents, and linear range of the 12 components are shown in Table 7. The correlation coefficient of standard curves of the 12 components showed they all have good linear correlation in the linear range. Catechin is the highest content component in the sample and its content reached 3.63%. The content of gallic acid is 0.15%, and it is the lowest content component. The total content of the 12 components is 11.18%.

## 4. Conclusions

In conclusion, the best extraction conditions of extraction yield, polyphenol content, antidiabetic activities, and antioxidant activity were optimized using response surface methodology, and BBD strategy demonstrated that it could be used for optimization of extraction process for KWZ. All of the optimization process and quantitative analysis should be useful for industrial production. Optimal conditions were found to be percentage of ethanol concentration 50.11%, extraction temperature 72.06°C, solid-to-solvent ratio 1 : 22.73 g/mL, and extraction time 2.93 h, which gave a maximum extraction yield of 39.28%, PTP-1B inhibition rate of 86.21%, *α*-glycosidase enzymes inhibition rate of 96.56%, and ABTS inhibition rate of 77.38%. Furthermore, 12 components were discovered on the effective parts with UPLC analysis, and the quantitative determination shows the total content of the 12 components is 11.18%. This optimal condition and quantitative analysis contribute to the better utilization of the prescription with high antidiabetic activity and antioxidant activity and are also helpful for industrial production improvement.

## Supplementary Material

All of the additional experimental data, including standard curve of 12 compounds (Figures S1–S12), screening test results of different proportion (Table S1) and the results of single-factor experiment (Tables S2–S6) can be found in supplementary materials.

## Figures and Tables

**Table 1:** Coded and uncoded levels of independent variables used in the RSM design.

Levels of coded variables	Independent variables			
	Ethanol concentration, % (*X*_1_)	Temperature, °C (*X*_2_)	Sample-to-solvent ratio, w/v (*X*_3_)	Extraction time, h (*X*_4_)
−1	30	60	1 : 10	2
0	50	70	1 : 20	3
1	70	80	1 : 30	4

**Table 2:** BBD and the observed responses for the independent variables of extraction conditions.

Run	Independent variables				Responses				
	*X*_1_	*X*_2_	*X*_3_	*X*_4_	*Y*_1_	*Y*_2_	*Y*_3_	*Y*_4_	*Y*_5_
1	50 (0)	80 (1)	20 (0)	4 (1)	35.88	45.24	79.24	77.54	69.14
2	50 (0)	80 (1)	10 (−1)	3 (0)	34.02	43.07	85.49	87.71	57.76
3	50 (0)	80 (1)	20 (0)	2 (−1)	38.58	47.4	67.66	77.81	68.52
4	50 (0)	60 (1)	20 (0)	2 (−1)	33.8	47.33	71.93	87.45	67.17
5	30 (−1)	70 (0)	20 (0)	4 (1)	35.22	43.53	80.34	98.43	67.54
6	70 (1)	80 (1)	20 (0)	3 (0)	35.75	47.05	82.62	98.17	76.91
7	50 (0)	70 (0)	20 (0)	3 (0)	39.14	47.08	87.93	94.12	75.58
8	70 (1)	70 (0)	20 (0)	2 (−1)	32.88	47.36	69.25	98.10	71.22
9	30 (−1)	70 (0)	30 (1)	3 (0)	37.96	46.01	72.72	93.77	66.06
10	50 (0)	70 (0)	30 (1)	2 (−1)	40.74	45.89	75.41	83.32	67.78
11	50 (0)	60 (1)	30 (1)	3 (0)	33.5	48.05	81.25	94.12	55.17
12	30 (−1)	70 (0)	20 (0)	2 (−1)	38.71	46.96	67.13	81.41	77.12
13	50 (0)	70 (0)	20 (0)	3 (0)	39.27	48.65	87.28	98.25	77.72
14	50 (0)	80 (1)	30 (1)	3 (0)	39.13	41.76	82.16	91.69	67.75
15	70 (1)	60 (1)	20 (0)	3 (0)	33.41	47.73	73.82	78.36	55.70
16	30 (−1)	70 (0)	10 (−1)	3 (0)	27.88	35.99	85.25	98.71	64.39
17	70 (1)	70 (0)	30 (1)	3 (0)	35.45	41.21	74.28	98.65	82.37
18	50 (0)	70 (0)	10 (−1)	2 (−1)	30.88	35.84	68.02	97.72	72.73
19	70 (1)	70 (0)	20 (0)	4 (1)	34.49	44.47	71.83	88.23	77.84
20	50 (0)	70 (0)	20 (0)	3 (0)	38.03	47.99	88.05	98.02	75.03
21	30 (−1)	60 (1)	20 (0)	3 (0)	34.57	48.23	78.41	98.69	65.58
22	50 (0)	60 (1)	10 (−1)	3 (0)	29.83	37.74	78.82	91.81	51.10
23	50 (0)	60 (1)	20 (0)	4 (1)	36.6	45.53	66.04	93.98	55.45
24	50 (0)	70 (0)	20 (0)	3 (0)	38.88	48.04	86.05	96.89	77.13
25	50 (0)	70 (0)	10 (−1)	4 (1)	30.24	40.41	83.20	94.92	57.53
26	50 (0)	70 (0)	30 (1)	4 (1)	40.35	36.95	67.15	97.40	79.90
27	30 (−1)	80 (1)	20 (0)	3 (0)	36.15	45.05	87.74	75.61	52.48
28	50 (0)	70 (0)	20 (0)	3 (0)	39.37	46.7	81.27	95.87	74.11
29	70 (1)	70 (0)	10 (−1)	3 (0)	29.27	41.22	79.06	97.58	59.85

*X*_1_, ethanol concentration (%); *X*_2_, temperature (°C); *X*_3_, Sample-to-solvent ratio (w/v); *X*_4_, extraction time (h); *Y*_1_, yield of extraction (%); *Y*_2_, total polyphenol content (%); *Y*_3_, PTP-1B inhibition rate (the sample concentration is 5 *μ*g/mL, %); *Y*_4_, *α*-glucosidase inhibition rate (the sample concentration is 50 *μ*g/mL, %); *Y*_5_, ABTS inhibition rate (the sample concentration is 8 *μ*g/mL, %).

**Table 3:** Analysis of variance.

Source	Sum of squares	Degrees of freedom	Mean of square	*F* value	*P* value
Yield of extraction					

Model	321.42	14	22.96	10.37	<0.0001 (significant)
Residual	31.00	14	2.21		
Pure error	1.17	4	0.29		
Lack of fit	29.83	10	2.98	10.24	0.0192
Total	352.41	28			
*R* ^2^	0.9120		Adj. *R*^2^	0.8241	

Model equation *Y*_1_ = 38.94 − 0.77*X*_1_ + 1.48*X*_2_ + 3.75*X*_3_ − 0.23*X*_4_ + 0.19*X*_1_*X*_2_ − 0.97*X*_1_*X*_3_ + 1.28*X*_1_*X*_4_ + 0.36*X*_2_*X*_3_ − 1.38*X*_2_*X*_4_ + 0.063*X*_3_*X*_4_ − 2.81*X*_1_^2^ − 1.62*X*_2_^2^ − 3.12*X*_3_^2^ − 0.73*X*_4_^2^					

*Total polyphenol content*					

Model	423.50	14	30.25	47.38	<0.0001 (significant)
Residual	8.94	14	0.64		
Pure error	2.49	4	0.62		
Lack of fit	6.45	10	0.65	1.04	0.5314 (not significant)
Total	432.42	28			
*R* ^2^	0.9793		Adj. *R*^2^	0.9587	

Model equation *Y*_2_ = 47.69 + 0.27*X*_1_ − 0.42*X*_2_ + 2.13*X*_3_ − 1.22*X*_4_ + 0.62*X*_1_*X*_2_ − 2.51*X*_1_*X*_3_ + 0.13*X*_1_*X*_4_ − 2.90*X*_2_*X*_3_ − 0.090*X*_2_*X*_4_ − 3.38*X*_3_*X*_4_ − 0.75*X*_1_^2^ + 0.43*X*_2_^2^ − 5.83*X*_3_^2^ − 1.73*X*_4_^2^					

*PTP-1B inhibition rate*					

Model	1318.42	14	94.17	9.82	<0.0001 (significant)
Residual	134.24	14	4.41		
Pure error	31.82	4	7.95		
Lack of fit	102.42	10	10.24	1.29	0.4350 (not significant)
Total	1452.66	28			
*R* ^2^	0.9076		Adj. *R*^2^	0.8152	

Model equation *Y*_3_ = 86.12 − 1.73*X*_1_ + 2.89*X*_2_ − 2.24*X*_3_ + 2.37*X*_4_ − 0.13*X*_1_*X*_2_ + 1.94*X*_1_*X*_3_ − 2.66*X*_1_*X*_4_ − 1.44*X*_2_*X*_3_ + 4.37*X*_2_*X*_4_ − 5.86*X*_3_*X*_4_ − 3.95*X*_1_^2^ − 2.36*X*_2_^2^ − 2.66*X*_3_^2^ − 10.86*X*_4_^2^					

*α-Glucosidase inhibition rate*					

Model	1449.06	14	103.50	10.43	<0.0001 (significant)
Residual	138.87	14	9.92		
Pure error	11.50	4	2.88		
Lack of fit	127.37	10	12.74	4.43	0.0822 (not significant)
Total	1587.93	28			
*R* ^2^	0.9125		Adj. *R*^2^	0.8251	

Model equation *Y*_4_ = 96.63 + 1.04*X*_1_ − 2.99*X*_2_ − 0.79*X*_3_ + 2.06*X*_4_ + 10.73*X*_1_*X*_2_ + 1.50*X*_1_*X*_3_ − 6.73*X*_1_*X*_4_ + 0.42*X*_2_*X*_3_ − 1.70*X*_2_*X*_4_ + 4.42*X*_3_*X*_4_ − 0.98*X*_1_^2^ − 7.58*X*_2_^2^ − 1.73*X*_3_^2^ − 4.66*X*_4_^2^					

*ABTS inhibition rate*					

Model	2213.57	14	158.11	33.37	<0.0001 (significant)
Residual	66.33	14	4.74		
Pure error	8.87	4	2.22		
Lack of fit	57.46	10	5.75	2.59	0.1859 (not significant)
Total	2279.90	28			
*R* ^2^	0.9709		Adj. *R*^2^	0.9418	

Model equation *Y*_5_ = 75.91 + 2.73*X*_1_ + 3.70*X*_2_ + 4.64*X*_3_ − 1.43*X*_4_ + 8.08*X*_1_*X*_2_ + 5.21*X*_1_*X*_3_ + 4.05*X*_1_*X*_4_ + 1.48*X*_2_*X*_3_ + 3.08*X*_2_*X*_4_ + 6.83*X*_3_*X*_4_ − 2.12*X*_1_^2^ − 11.41*X*_2_^2^ − 6.20*X*_3_^2^ − 7.65*X*_4_^2^					

Value of lack of fit as analyzed by ANOVA.

**Table 4:** Predicted values for the response variables.

Response variables	*X*_1_	*X*_2_	*X*_3_	*X*_4_	Predicted values
Yield of extraction (%)	48.80	74.29	25.09	3.04	40.84
Total polyphenol content (%)	50.11	72.06	22.73	2.93	48.44
PTP-1B inhibition rate (%)	52.10	73.80	21.84	2.95	86.21
*α*-glucosidase inhibition rate (%)	47.57	69.74	22.22	3.03	96.56
ABTS inhibition rate (%)	52.70	71.50	22.38	3.01	77.68

*X*_1_, ethanol concentration (%); *X*_2_, temperature (°C); *X*_3_, sample-to-solvent ratio (w/v); *X*_4_, extraction time (h).

**Table 5:** DPPH radical scavenging activity of 29 design experiments.

Run	DPPH IR (%)
1	83.90 ± 0.35
2	78.88 ± 0.47
3	95.73 ± 0.36
4	83.22 ± 0.45
5	96.60 ± 0.35
6	78.71 ± 0.41
7	94.35 ± 0.36
8	92.18 ± 0.35
9	96.63 ± 0.35
10	97.39 ± 0.35
11	83.68 ± 0.51
12	97.65 ± 0.35
13	93.41 ± 0.38
14	90.68 ± 0.40
15	91.15 ± 0.35
16	86.99 ± 0.43
17	86.36 ± 0.40
18	83.28 ± 0.41
19	89.24 ± 0.36
20	94.35 ± 0.35
21	86.52 ± 0.50
22	84.97 ± 0.51
23	95.50 ± 0.35
24	93.92 ± 0.35
25	90.21 ± 0.39
26	86.92 ± 0.40
27	93.65 ± 0.39
28	92.42 ± 0.38
29	87.20 ± 0.45
*V*_*c*_	88.98 ± 0.42

Data are expressed as mean ± SD (*n* = 3); IR is the inhibition rate of extracts (the sample concentration is 12.5 *μ*g/mL).

**Table 6:** Analytical results of precision, stability, and repeatability test of 12 peaks in the KWZ (*n* = 6).

Peak number	RSD of RRT (%)			RSD of RPA (%)		
	Precision	Stability	Repeatability	Precision	Stability	Repeatability
1	0.17	0.23	0.30	0.65	1.00	1.10
2	0.18	0.17	0.14	1.24	1.11	1.24
3	0.08	0.09	0.21	1.72	1.77	1.74
4	0.03	0.03	0.03	1.15	1.85	1.35
5	0.00	0.00	0.00	0.00	0.00	0.00
6	0.08	0.12	0.11	1.55	1.55	1.65
7	0.04	0.08	0.05	0.82	0.94	0.86
8	0.05	0.08	0.06	0.87	0.72	0.57
9	0.04	0.08	0.06	0.80	0.72	0.60
10	0.04	0.06	0.05	1.08	0.92	0.71
11	0.06	0.07	0.05	0.85	1.39	1.23
12	0.06	0.05	0.04	1.37	1.49	1.63

**Table 7:** Retention time, name, regression equation, contents, and linear range of 12 components in the purified prescription.

Number	*t*_*R*_ (min)	Component	Regression equation	*R* ^2^	Content (%)	Linear range (*μ*g/mL)
1	2.4	Gallic acid	*y* = 6649.1*x* − 625.98	0.9999	0.15	10.0–70.0
2	10.5	Catechin	*y* = 558.67*x* + 3342.2	0.9999	3.63	20.0–1100.0
3	13.0	Chlorogenic acid	*y* = 4932.2*x* − 11477	1.0000	0.48	57.5–287.0
4	16.8	Epicatechin	*y* = 708.8*x* + 2911.6	0.9999	0.36	45.0–225.0
5	18.2	Corilagin	*y* = 3623.1*x* + 1735.8	1.0000	1.97	130.0–650.0
6	40.9	Ellagic acid	*y* = 21560*x* − 345092	0.9965	0.45	65.0–228.0
7	53.3	Hyperoside	*y* = 7500.7*x* + 2857.1	1.0000	1.11	55.0–330.0
8	53.9	Rutin	*y* = 7143.9*x* + 4530.4	0.9999	1.11	57.5–345.0
9	54.8	Isoquercitrin	*y* = 9655.1*x* − 12230	1.0000	0.87	44.0–308.0
10	59.8	Avicularin	*y* = 12645*x* − 11462	0.9995	0.22	30.0–150.0
11	63.9	Quercitrin	*y* = 16511*x* − 20026	0.9998	0.31	30.0–150.0
12	80.9	Quercetin	*y* = 2568.9*x* − 12928	0.9992	0.51	45.0–225.0
