# Supplementary material for: Optimization of Extraction Process for Antidiabetic and Antioxidant Activities of Kursi Wufarikun Ziyabit Using Response Surface Methodology and Quantitative Analysis of Main Components
Source: Evid Based Complement Alternat Med. 2017 May 17;2017:6761719. doi: 10.1155/2017/6761719 (PMC5450171; doi:10.1155/2017/6761719)
Supplement: Supplementary file 1 — All of the additional experimental data, including standard curve of 12 compounds (Figures S1–S12), screening test results of different proportion (Table S1) and the results of single-factor experiment (Tables S2–S6) can be found in supplementary materials. [file 6761719.f1.pdf]

Optimization of Extraction Process for Anti-diabetic and Antioxidant Activities of *Kursi Wufarikun Ziyabit* Using Response Surface Methodology and Quantitative Analysis of Main Components

Salamet Edirs [1,2,3], Ablajan Turak [1,2], Sodik Numonov[1,2, 4], XueLei Xin [1,2,*]and Haji Akber Aisa [1,2,*]

[1] The Key Laboratory of Plant Resources and Chemistry of Arid Zone, Xinjiang Technical Institute of Physics and Chemistry, Chinese Academy of Sciences, Urumqi 830011, China.

[2] State Key Laboratory Basis of Xinjiang Indigenous Medicinal Plants Resource Utilization, Xinjiang Technical Institute of Physics and Chemistry, Chinese Academy of Sciences, Urumqi 830011, China.

[3] University of the Chinese Academy of Sciences, Beijing 100039, China.

[4] State Scientifically-Experimental and Production Organization, Academy of Sciences of the Republic of Tajikistan, Dushanbe 734063, Tajikistan.

* Corresponding authors: haji@ms.xjb.ac.cn;
xinxl@ms.xjb.ac.cn;

Contents:

Fig S1 Standard curve of Gallic acid

Fig S2 Standard curve of Catechin

Fig S3 Standard curve of Chlorogenic acid

Fig S4 Standard curve of Epicatechin

Fig S5 Standard curve of Corilagin

Fig S6 Standard curve of Ellagic acid

Fig S7 Standard curve of Hyperoside

Fig S8 Standard curve of Rutin

Fig S9 Standard curve of Isoquercitrin

Fig S10 Standard curve of Avicularin

Fig S11 Standard curve of Quercitrin

Fig S12 Standard curve of Quercetin

Table S1 Screening test results of different proportion

Table S2 The influence of ethanol concentration to extraction

Table S3 The influence of temperature to extraction

Table S4 The influence of sample to solvent ratio to extraction

Table S5 The influence of extraction time to extraction

Table S6 The influence of extraction times to extraction

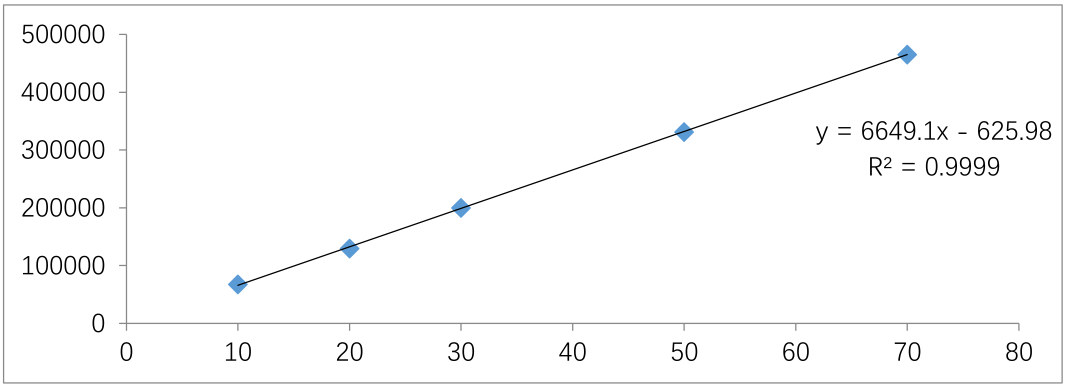

Fig S1 Standard curve of Gallic acid

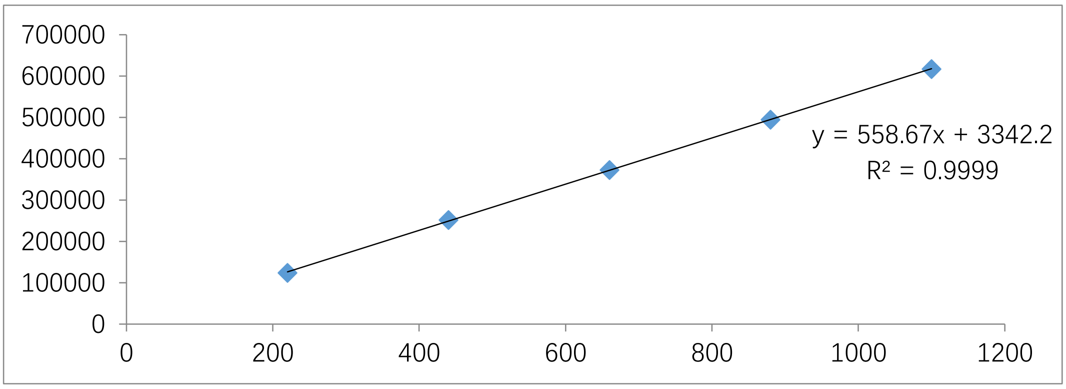

Fig S2 Standard curve of Catechin

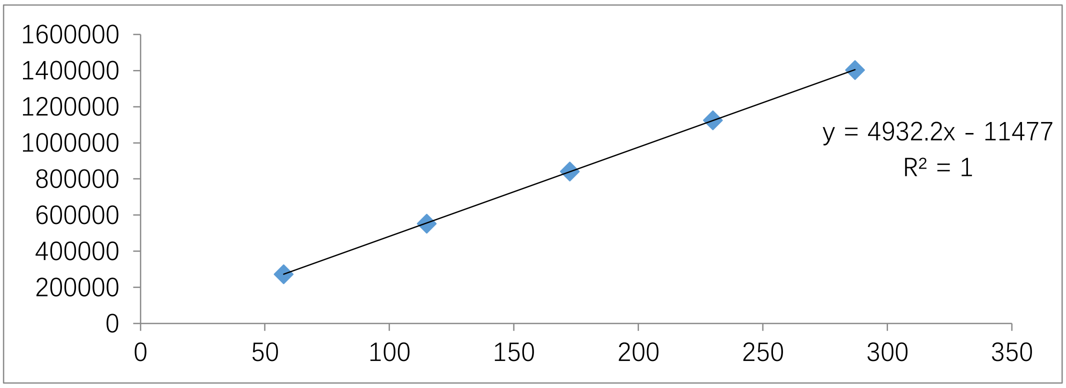

Fig S3 Standard curve of Chlorogenic acid

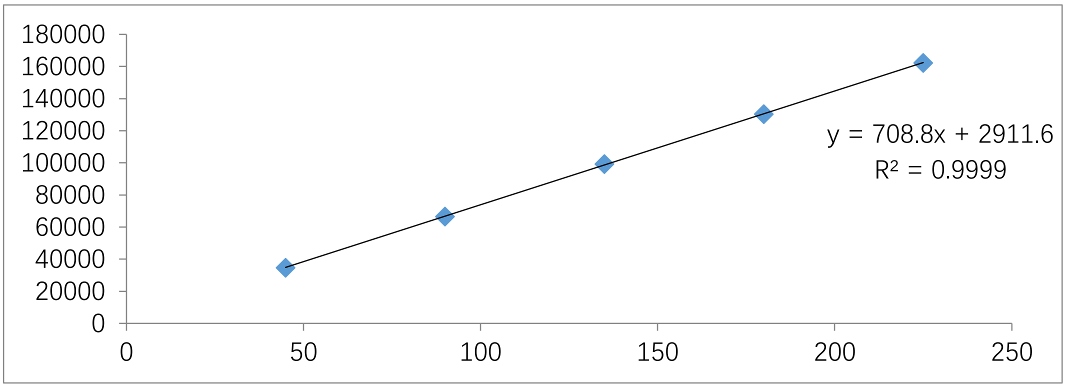

Fig S4 Standard curve of Epicatechin

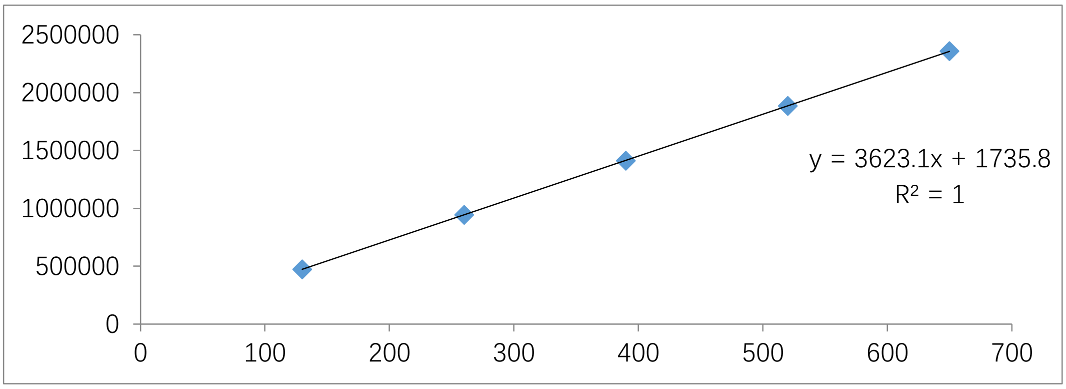

Fig S5 Standard curve of Corilagin

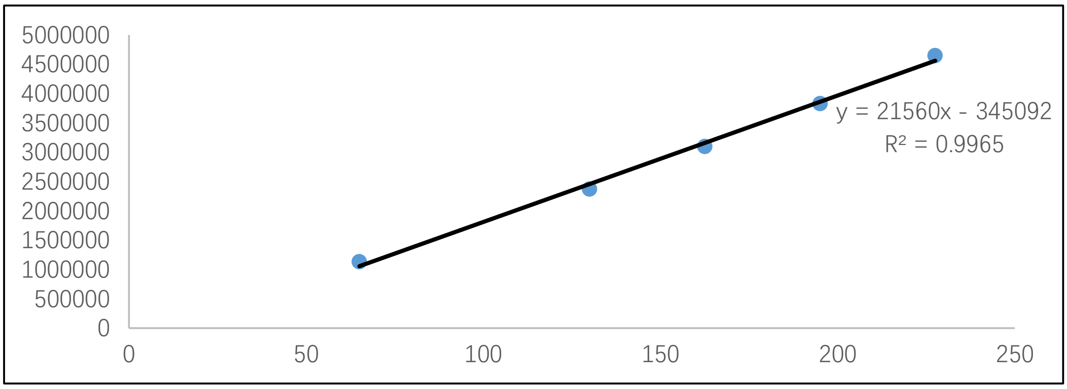

Fig S6 Standard curve of Ellagic acid

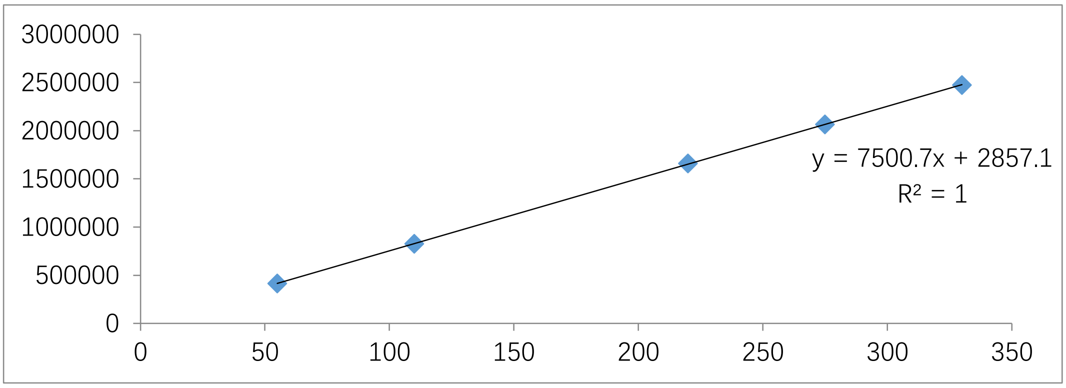

Fig S7 Standard curve of Hyperoside

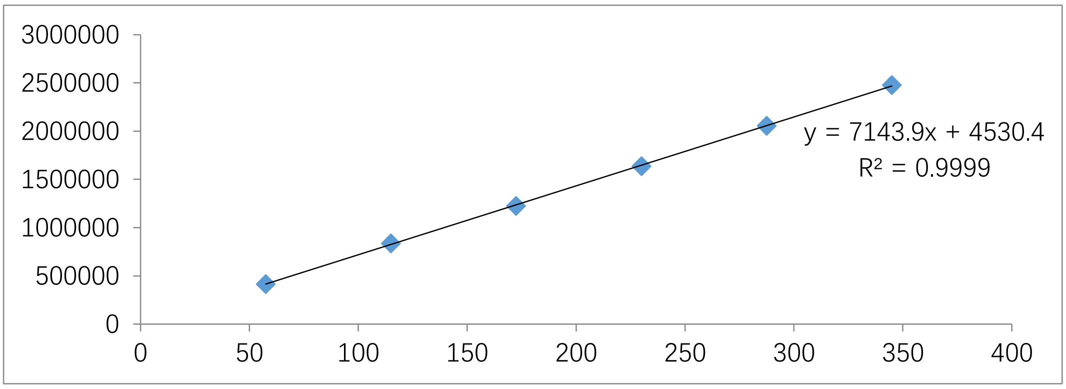

Fig S8 Standard curve of Rutin

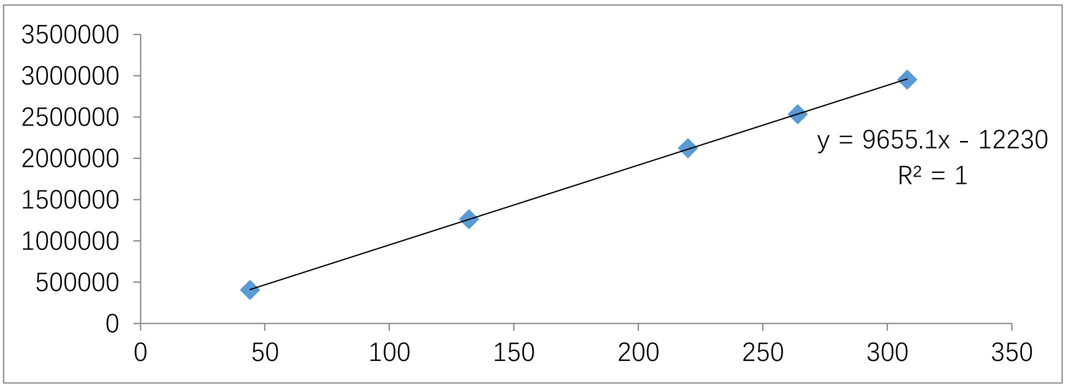

Fig S9 Standard curve of Isoquercitrin

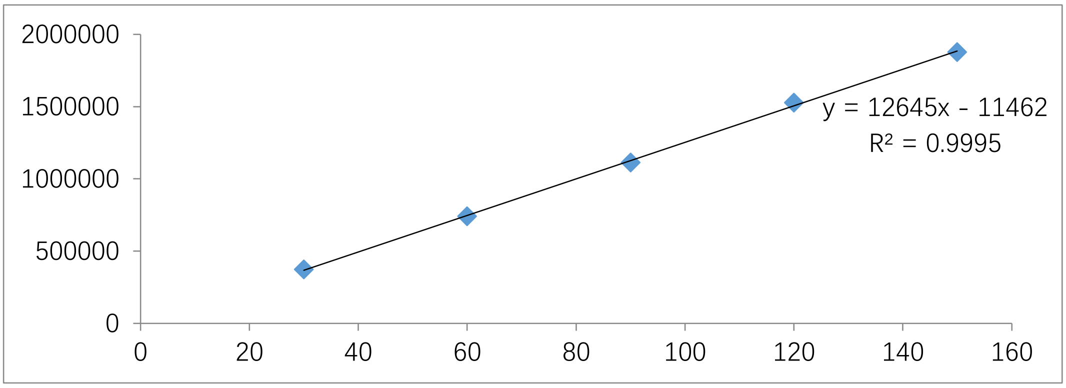

Fig S10 Standard curve of Avicularin

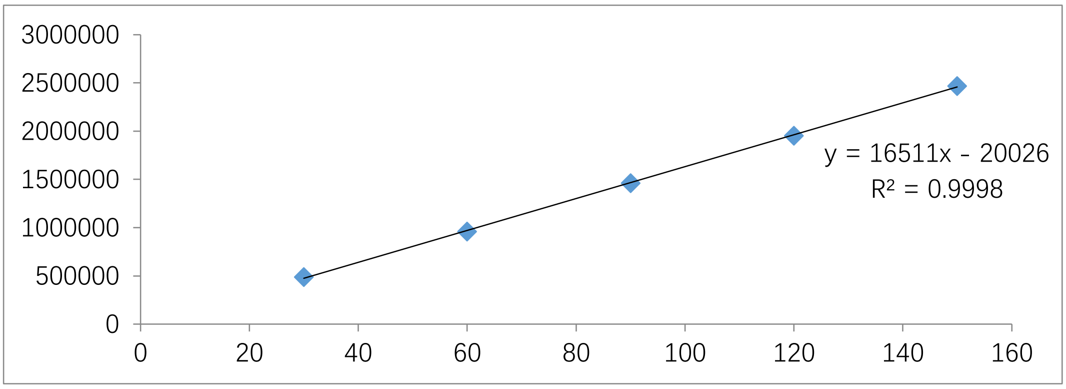

Fig S11 Standard curve of Quercitrin

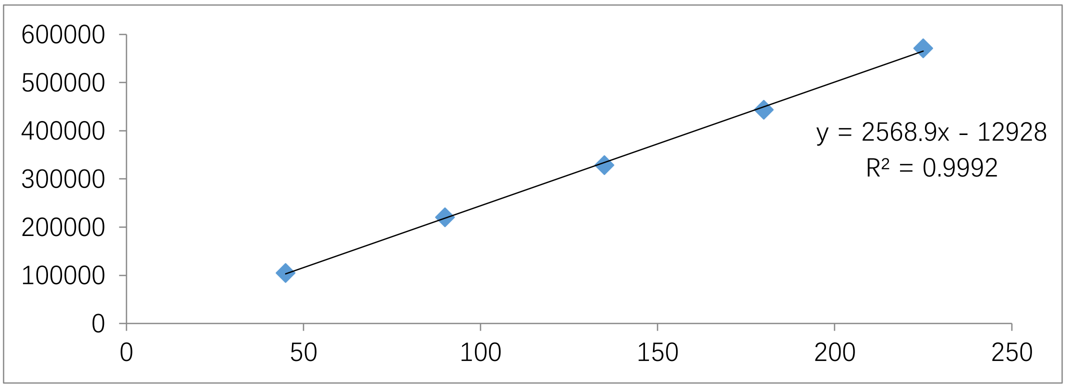

Fig S12 Standard curve of Quercetin

Table S1 Screening test results of different proportion

**Ratio**	**EY (%)**	**TPC (%)**	**PTP-1B IC50 (μg/mL)**	**α-Glu IC50 (μg/mL)**	**ABTS IC50 (μg/mL)**
0:1	32.62	28.70	2.82±0.31	42.21±0.56	8.44±0.63
1:1	34.57	38.90	1.42±0.35	7.70±0.61	4.40±0.52
2:8	37.82	30.51	2.54±0.45	16.77±0.46	7.37±0.58
3:7	32.10	31.01	2.03±0.35	14.41±0.58	10.14±0.51
4:6	34.25	34.61	1.56±0.37	7.35±0.40	8.63±0.62
6:4	35.52	31.43	1.04±0.35	5.00±0.49	3.02±0.32
7:3	36.18	39.60	0.19±0.35*	1.04±0.45*	1.11±0.32*
8:2	38.54*	34.24	0.35±0.44	2.19±0.45	1.23±0.32
9:1	37.40	36.30	0.33±0.52	1.37±0.32	1.15±0.33
1:0	30.66	46.88*	1.06±0.35	1.81±0.37	3.32±0.42

EY: Extraction Yield;

TPC: Total polyphenol content;

*: show the best results

Table S2 The influence of ethanol concentration to extraction

Ethanol concentration (%)	EY (%)	PTP-1B IC50 (μg/mL)	TPC (%)	Comprehensive score
10	13.56	1.91±0.35	23.94	72.41
30	11.45	1.50±0.34	29.83	82.79
50	17.65	1.65±0.35	38.14	91.41
70	10.65	1.34±0.39	29.12	82.41
90	11.38	1.69±0.37	28.95	76.45
absolute ethanol	11.65	1.29±0.33	27.44	86.53

Comprehensive score including extraction yield (EY) 30%+total polyphenol content (TPC) 30%+PTP-1B activity 40%

Table S3 The influence of temperature to extraction

Temperature (º C)	EY (%)	PTP-1B IC50 (μg/mL)	TPC (%)	Comprehensive score
40	12.28	3.38±0.30	45.54	68.16
50	12.63	1.78±0.30	44.71	69.6
60	13.48	1.63±0.34	38.07	73.21
70	13.29	0.99±0.37	49.07	94.98
80	13.07	1.09±0.33	46.92	80.15

Comprehensive score including extraction yield (EY) 30%+total polyphenol content (TPC) 30%+PTP-1B activity 40%

Table S4 The influence of sample to solvent ratio to extraction

Sample to solvent (W/V, g/mL)	EY（%）	PTP-1B IC50 (μg/mL)	TPC（%）	Comprehensive score
1:10	10.27	0.52±0.43	43.08	86.26
1:20	14.50	0.32±0.33	38.00	92.08
1:30	16.98	0.44±0.45	37.48	91.63

Comprehensive score including extraction yield (EY) 30%+total polyphenol content (TPC) 30%+PTP-1B activity 40%

Table S5 The influence of extraction time to extraction

Extraction time (h)	EY（%）	PTP-1B IC50 (μg/mL)	TPC（%）	Comprehensive score
1	14.65	0.71±0.40	41.07	91.99
2	16.88	0.66±0.32	38.06	89.90
3	17.34	0.58±0.46	41.11	97.11
4	17.49	0.93±0.36	45.06	96.33

Comprehensive score including extraction yield (EY) 30%+total polyphenol content (TPC) 30%+PTP-1B activity 40%

Table S6 The influence of extraction times to extraction

Extraction times (n)	EY（%）	PTP-1B IC50 (μg/mL)	TPC（%）	Comprehensive score
1	12.21	0.31±0.39	37.29	74.44
2	29.64	0.28±0.39	39.39	89.47
3	37.79	0.31±0.39	43.79	99.93
4	37.88	1.09±0.33	34.44	92.99

Comprehensive score including extraction yield (EY) 30%+total polyphenol content (TPC) 30%+PTP-1B activity 40%
